# Evaluation of the efficacy and safety of amustaline/glutathione pathogen-reduced RBCs in complex cardiac surgery: the Red Cell Pathogen Inactivation (ReCePI) study—protocol for a phase 3, randomized, controlled trial

**DOI:** 10.1186/s13063-023-07831-x

**Published:** 2023-12-11

**Authors:** Edward L. Snyder, Michael E. Sekela, Ian J. Welsby, Yoshiya Toyoda, Mohamed Alsammak, Neel R. Sodha, Thomas M. Beaver, J. Peter R. Pelletier, James D. Gorham, John S. McNeil, Roman M. Sniecinski, Ronald G. Pearl, Gregory A. Nuttall, Ravi Sarode, T. Brett Reece, Alesia Kaplan, Robertson D. Davenport, Tina S. Ipe, Peyman Benharash, Ileana Lopez-Plaza, Richard R. Gammon, Patrick Sadler, John P. Pitman, Kathy Liu, Stanley Bentow, Laurence Corash, Nina Mufti, Jeanne Varrone, Richard J. Benjamin, for the ReCePI study group

**Affiliations:** 1 Yale University School of Medicine, New Haven, CT USA; 2 Gill Heart Institute University of Kentucky, Lexington, KY USA; 3 Duke University Medical Center, Durham, NC USA; 4 Temple University Hospital, Philadelphia, PA USA; 5 Temple University Health System, Philadelphia, PA USA; 6 Rhode Island Hospital, Providence, RI USA; 7 University of Florida, Gainesville, FL USA; 8 University of Pittsburgh Medical Center, Pittsburgh, PA USA; 9 Emory University, Atlanta, GA USA; 10 University of Colorado Hospital, Denver, CO USA; 11 Stanford University, Stanford, CA USA; 12 University of Michigan, Ann Arbor, MI USA; 13 University of Virginia Health System, Charlottesville, VA USA; 14 University of Texas, Southwestern, Dallas, TX USA; 15 Our Blood Institute, Oklahoma City, OK USA; 16 University of Arkansas for Medical Sciences, Little Rock, AR USA; 17 Mayo Clinic, Rochester, MN USA; 18 UCLA, Los Angeles, CA USA; 19 Henry Ford Hospital, Detroit, MI USA; 20 Scientific, Medical and Technical and Research Department, OneBlood, Orlando, FL USA; 21 Central California Blood Center, Fresno, CA USA; 22 Cerus Corporation, 1220 Concord Ave, Concord, CA 94520 USA; 23 Vitalant, Pittsburgh, PA USA

**Keywords:** Amustaline/GSH, INTERCEPT, Pathogen reduction, Transfusion-transmitted infections, Randomized controlled trial, Cardiac surgery, Acute kidney injury

## Abstract

**Background:**

Red blood cell (RBC) transfusion is a critical supportive therapy in cardiovascular surgery (CVS). Donor selection and testing have reduced the risk of transfusion-transmitted infections; however, risks remain from bacteria, emerging viruses, pathogens for which testing is not performed and from residual donor leukocytes. Amustaline (S-303)/glutathione (GSH) treatment pathogen reduction technology is designed to inactivate a broad spectrum of infectious agents and leukocytes in RBC concentrates. The ReCePI study is a Phase 3 clinical trial designed to evaluate the efficacy and safety of pathogen-reduced RBCs transfused for acute anemia in CVS compared to conventional RBCs, and to assess the clinical significance of treatment-emergent RBC antibodies.

**Methods:**

ReCePI is a prospective, multicenter, randomized, double-blinded, active-controlled, parallel-design, non-inferiority study. Eligible subjects will be randomized up to 7 days before surgery to receive either leukoreduced Test (pathogen reduced) or Control (conventional) RBCs from surgery up to day 7 post-surgery. The primary efficacy endpoint is the proportion of patients transfused with at least one study transfusion with an acute kidney injury (AKI) diagnosis defined as any increased serum creatinine (sCr) level ≥ 0.3 mg/dL (or 26.5 µmol/L) from pre-surgery baseline within 48 ± 4 h of the end of surgery. The primary safety endpoints are the proportion of patients with any treatment-emergent adverse events (TEAEs) related to study RBC transfusion through 28 days, and the proportion of patients with treatment-emergent antibodies with confirmed specificity to pathogen-reduced RBCs through 75 days after the last study transfusion. With ≥ 292 evaluable, transfused patients (> 146 per arm), the study has 80% power to demonstrate non-inferiority, defined as a Test group AKI incidence increase of no more than 50% of the Control group rate, assuming a Control incidence of 30%.

**Discussion:**

RBCs are transfused to prevent tissue hypoxia caused by surgery-induced bleeding and anemia. AKI is a sensitive indicator of renal hypoxia and a novel endpoint for assessing RBC efficacy. The ReCePI study is intended to demonstrate the non-inferiority of pathogen-reduced RBCs to conventional RBCs in the support of renal tissue oxygenation due to acute anemia and to characterize the incidence of treatment-related antibodies to RBCs.

**Supplementary Information:**

The online version contains supplementary material available at 10.1186/s13063-023-07831-x.

## Introduction

The safety of the blood supply has improved markedly over the last 50 years [1]; however, transfusion-transmitted infections (TTI) caused by viruses, bacteria, and protozoa, as well as residual leukocytes causing transfusion-associated graft-versus-host disease (TA-GVHD) [2–4] still pose a threat to transfusion recipients [5]. Current strategies to prevent TTI and TA-GVHD include pre-donation evaluation and selection of blood donors with low-risk behavioral profiles, followed by serologic and/or nucleic acid testing for a limited number of known infectious pathogens, plus selective irradiation to prevent TA-GVHD. Pathogen reduction technologies (PRT) are now widely available to further reduce these risks with platelet and plasma transfusions [6] but are not yet commercially available for RBC transfusions.

To proactively address the risk of TTIs and TA-GVHD associated with RBC transfusions, Cerus Corporation (Concord, CA, USA) is developing a pathogen and leukocyte inactivation technology for RBCs (the INTERCEPT® Blood System for RBCs) under a contract with the US Department of Health and Human Services (DHHS) Biomedical Advanced Research and Development Authority (BARDA). The system uses amustaline, a nucleic acid-targeting molecule, to inactivate a broad spectrum of bacteria, viruses, protozoa, and leukocytes [7, 8]. Added within 24 h to packed leukoreduced RBCs separated from whole blood collections, amustaline forms irreversible adducts and covalent crosslinks within single- or double-stranded DNA or RNA to inhibit pathogen and leukocyte replication. Glutathione (GSH) is included to reduce unwanted side reactions with non-nucleic acid molecules [9]. Amustaline/GSH-treatment is designed to inactivate T-cells to prevent TA-GVHD [10], replacing irradiation. The pathogen reduction process includes a terminal media exchange step that further reduces the concentration of plasma proteins including antibodies, producing a final RBC product that meets the European Directorate for the Quality of Medicines & Healthcare (EDQM) standard for washed RBCs [11], which may reduce the incidence of allergic reactions and transfusion-related acute lung injury (TRALI). Pathogen-reduced RBCs may be stored for up to 35 days at 1–6°C under standard transfusion service conditions.

A robust method for pathogen reduction of RBCs would permit pathogen reduction to be implemented for all labile blood components prepared for transfusion (RBCs, plasma, and platelets), thereby reducing the overall residual risk to patients from TTIs associated with emerging and known pathogens and TA-GVHD. Increased safety margins with PRT may also allow for the reassessment of deferral criteria (e.g., malaria travel deferrals) and the potential expansion of blood donor pools; may preclude the addition of future tests for emerging infectious diseases; eliminate existing tests to reduce cost; and permit the replacement of irradiation for the prevention of TA-GVHD.

The INTERCEPT Blood System for RBCs is in development, and in vitro studies have demonstrated inactivation of a wide range of bacterial species, enveloped and non-enveloped viruses, protozoa, and leukocytes [7, 9, 12]. The relative infrequency of each of these threats in most countries’ blood supplies precludes the execution of clinical studies to demonstrate reduction of TTI and TA-GVHD risk (changes in these risk patterns will be described in post-market studies, as has been done with pathogen-reduced platelets) [6, 13, 14]. Therefore, clinical studies with amustaline/GSH RBCs have focused on demonstrating that pathogen-reduced RBCs retain physiologic function in vitro and survival in vivo, with a safety profile comparable to conventional RBCs.

Amustaline/GSH RBCs demonstrated adequate viability with in vivo recovery and lifespan studies [15]. Two completed Phase 3 clinical studies conducted in CVS [16] and β-thalassemia patients [17] in Europe demonstrated that pathogen-reduced RBCs possess in vitro and in vivo characteristics comparable to conventional (untreated) RBCs, with a similar safety profile [18]. A third Phase 3 trial [the RedeS trial, www.clinicaltrials.gov ID: NCT03037164] is underway with pathogen-reduced RBCs in the USA in patients requiring transfusion for a broad array of indications, including chronically transfused sickle cell disease (SCD) patients.

## Methods/design

CLI 00125: A Randomized, Double-Blinded, Controlled, Parallel Group, Non-inferiority, Phase III Study to Evaluate the Efficacy and Safety of the INTERCEPT Blood System for Red Blood Cells in Patients undergoing Complex Cardiac Surgery Procedures (hereafter referred to as the ReCePI study) is designed to demonstrate that amustaline/GSH RBCs are non-inferior to conventional RBCs with regard to clinical safety and efficacy when transfused for acute anemia in patients undergoing complex CVS with potential hemodynamic instability. In addition, since natural antibodies specific for amustaline/GSH RBCs have been described in patients never exposed to these RBCs [8, 19], and treatment-emergent antibodies have been described in earlier clinical studies with a prior iteration of the amustaline/GSH pathogen reduction technology, the ReCePI study is designed to evaluate the incidence, nature, and clinical significance [20] of treatment-emergent antibodies specific for amustaline/GSH pathogen-reduced RBCs.

The clinical outcome of renal impairment will be used to determine the non-inferiority of pathogen-reduced RBCs compared to conventional RBCs. ReCePI is sponsored by Cerus Corporation (hereafter referred to as the Sponsor). The trial will be conducted according to the protocol subject to institutional review board (IRB) approval, the International Council on Harmonization E6 Good Clinical Practice (GCP), and applicable local/national regulatory requirements and laws. This report has been formatted using the SPIRIT reporting guidelines [21], as described in the SPIRIT checklist provided in the supplemental materials.

### Participants

Subjects will be recruited in hospital or outpatient settings at 18 US-based centers where complex cardiac surgeries are routinely performed. Study sites are selected based on the availability of potentially suitable subjects and the investigators’ access to those subjects.

The target patient population is age ≥ 11 years and ≥ 40 kg in weight, scheduled for complex cardiac surgery or thoracic aorta surgery with a high probability of needing RBC transfusion. The planned procedure may be performed with or without cardiopulmonary bypass (CPB) and/or cell salvage. Informed consent will be obtained by the site investigators or by authorized and trained study staff and will include consent for publication of anonymized data and study outcomes. Any unplanned ancillary studies with patients’ biological specimens or data will require additional consent. To minimize enrollment of patients who do not receive a RBC transfusion, only patients with a high probability of RBC transfusion during or after surgery (defined by a TRUST score of ≥ 3 [22]), or currently on a regimen of aspirin, clopidogrel, or analogs and/or GPIIb/IIIa inhibitors, or at the discretion of the investigator, will be enrolled. Female subjects of child-bearing potential must be using appropriate birth control during the study. Patients will be excluded prior to randomization if they meet any of the criteria shown in Table 1.

**Table 1:** Exclusion criteria

Patients will be excluded if they meet any of the following criteria prior to randomization:
1. Confirmed positive baseline serum/plasma antibody specific for amustaline/GSH-treated RBCs
2. Pregnant or breast feeding
3. Refusal of blood products or other inability to comply with the protocol in the opinion of the investigator or the treating physician
4. Treatment with any medication that is known to adversely affect RBC viability, such as, but not limited to, dapsone, levodopa, methyldopa, nitrofurantoin, and its derivatives, phenazopyridine and quinidine
5. Planned cardiac transplantation
6. Left ventricular assist device (LVAD) or extracorporeal membrane oxygenation (ECMO) support pre-operatively or a planned need post-operatively
7. Cardiogenic shock requiring pre-operative placement of an intra-aortic balloon pump
8. Active autoimmune hemolytic anemia
9. Planned use of autologous or directed donations
10. RBC transfusion during current hospitalization within 7 days prior to randomization
11. Participation in an interventional clinical study concurrently or within the previous 28 days
12. Patients with a current diagnosis of either chronic kidney disease or acute kidney injury requiring RRT and/or with sCr ≥ 1.8 mg/dL at screening
13. Patients with a current diagnosis of either chronic or acute hepatic insufficiency and a total serum bilirubin ≥ 2.0 mg/dL (≥ 34.2 µmol/L)
14. Pre-existing RBC antibodies that make the provision of compatible study RBC components difficult, at the investigator’s discretion
15. History of TRs requiring washed RBCs, volume-reduced RBCs, or RBCs with additive solution removed
16. Patients with documented IgA deficiency or a history of severe allergic reactions to blood products
17. Patients who require irradiated RBC blood components
18. Positive DAT with a polyspecific DAT reaction strength > 2 + , or any polyspecific DAT with pan-reactivity with a commercial IAT antibody screening panel that precludes the identification of underlying alloantibodies or indicates the presence of autoantibody

### Interventions

The Test component is allogeneic leukocyte-reduced amustaline/GSH RBCs suspended and stored in SAG-M at 1 to 6 °C for up to 35 days post-donation. The comparator (Control) component is standard-of-care, leukocyte-reduced RBCs suspended in a Food and Drug Administration (FDA) approved additive solution (e.g., AS-1, AS-3 or AS-5) and stored at 1 to 6 °C for up to 35 days. Both Test and Control RBC components will be provided by the Sponsor. Study RBCs will be collected, produced, and labeled by one of four blood centers equipped, trained and funded by the Sponsor to produce amustaline/GSH and conventional RBCs with appropriate labeling to ensure that study investigators will remain blinded. Transfusion services at participating study hospitals will not be blinded to allow for efficient RBC stock management. Study RBCs (both Test and Control) will be labeled as investigational products and may not be used in routine care of non-study patients.

### Treatment plan

**Fig. 1:**
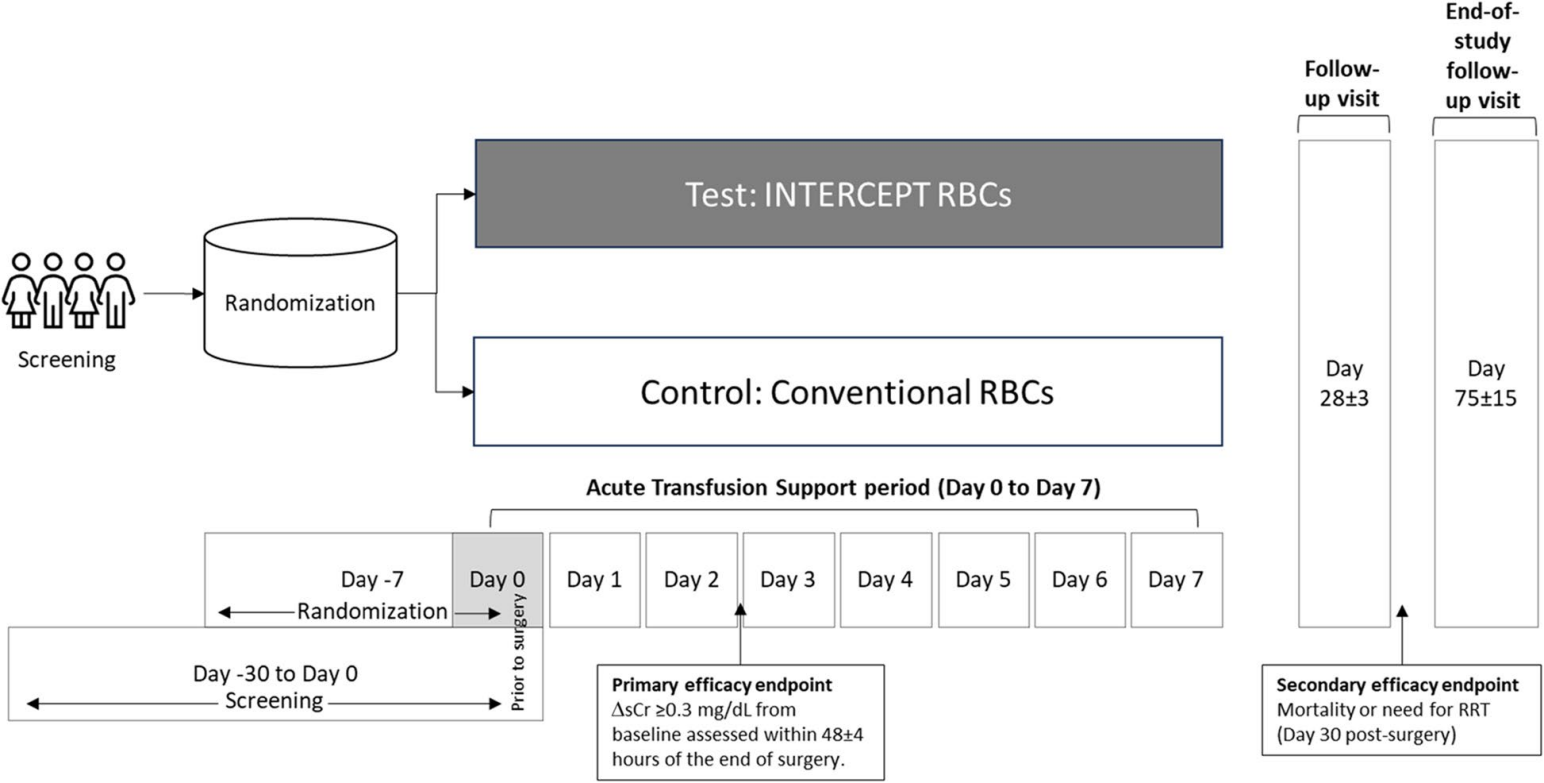
Study timeline

The study timeline is shown in Fig. 1. A summary of the study’s schedule of enrolment, interventions, and assessments is provided in Table 2.

**Table 2:** Schedule of Enrollment, Interventions, and Assessments

	**Study period**							
**Timepoint**	**Enrollment**	**Allocation**	**Post-allocation**					
			**Surgery**	**Acute transfusion period**	**Adverse event monitoring**	**End of monitoring period**	**Status**	**Close-out**
	**Day -30 to day 0 pre-surgery**	**Day -7 to day 0 pre-surgery**	**Day 0 surgery**	**Day 0 post-surgery–day 7**	**Day 8–28**	**Day 28**	**Day 30**	**Day 75**
**Enrolment**								
Informed consent	X							
Demographics & medical history	X							
Baseline lab studies	X							
S-303 antibody screening panel	X							
Baseline serum creatinine	X							
Sample for HLA antibodies	X							
Pre-transfusion testing	X							
Randomization		X						
**Intervention**								
Cardiac or thoracic aorta surgery			X					
Study RBC transfusions			X	X				
**Assessments**								
Serum creatinine			X	X (48 h)				
Document daily labs			X	X				
DAT						X		
Document AEs, SAEs and TRs			X	X	X	X		
S-303 antibody screening panel				(as needed)		X		X
Sample for HLA antibodies						X		
Vitals status and RRT status							X	X
Investigate S-303 antibodies				X	X	X	X	X
Document non-study blood components			X	X	X	X		

#### Screening/randomization (day − 30 to day 0 pre-surgery)

**Fig. 2:**
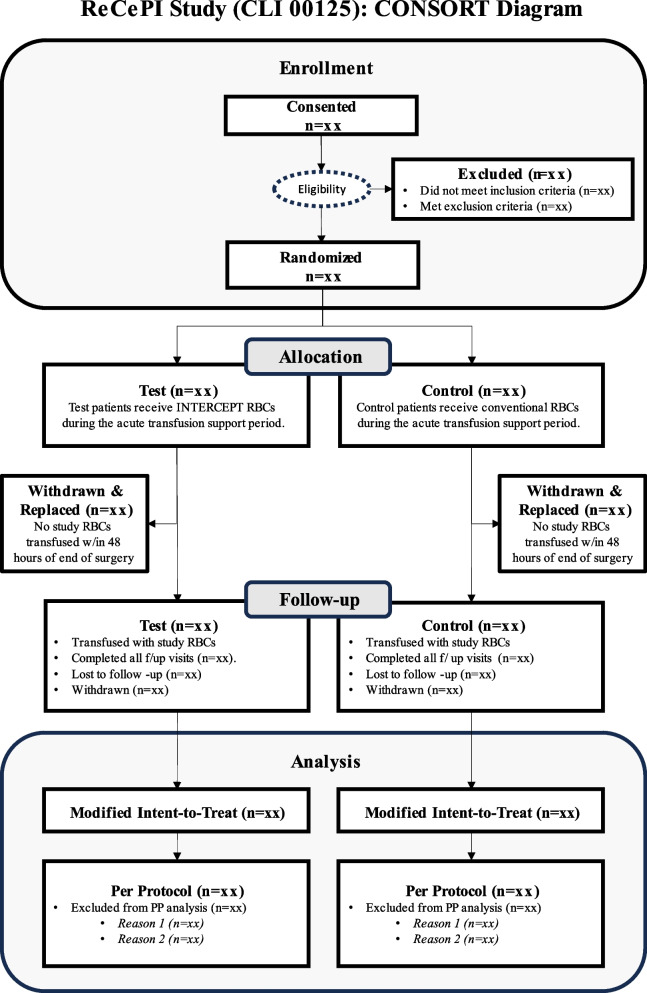
Consort diagram of study flow

Patients will be identified through pre-operative scheduling procedures in advance of planned surgery. Patients undergoing elective or urgent cardiac surgery are eligible for the study. Study consent/assent will be sought within 30 days of the surgical procedure (Fig. 2), including the day of surgery. Subjects who consent/assent to the study will be assigned a subject identification number and undergo screening. Screening data (e.g., inclusion/exclusion criteria) may be derived from the medical record when performed within 30 days of surgery. Blood samples for the determination of HLA antibodies will be collected and sent for testing at a specialized central laboratory. A screen for antibodies specific for amustaline/GSH RBCs will be performed using validated methods provided by the Sponsor at the local hospital transfusion service. Patients who fail eligibility for any inclusion/exclusion criteria may be rescreened for eligibility closer to the time of surgery. Eligible subjects may be randomized up to 7 days before or on the day of surgery, but prior to the start of surgery (i.e., induction of anesthesia).

An online Interactive Web Response System (IWRS) will be used by unblinded transfusion service personnel to randomize eligible patients. Unblinded transfusion service staff will also be responsible for the preparation and dispensing of study RBC components. Randomization (in a 1:1 ratio for Test: Control) will be stratified by site, pre-existing renal impairment (baseline sCr ≥ 1.2 mg/dL vs. < 1.2 mg/dL), and cardiac surgery group (more at risk for renal complications vs. less at risk).

#### Acute transfusion support period (surgery [day 0] to post-operative day 7, hospital discharge or death)

During the acute transfusion support period (day 0 to day 7, or until hospital discharge or death, whichever is first), patients will be transfused with Test or Control RBCs based on their randomization assignment. A screen for antibodies specific for amustaline/GSH RBCs will be performed every time a routine indirect antiglobulin test (IAT) is performed during the acute transfusion support period. Randomized subjects who do not receive a study RBC transfusion within the first 48 h after surgery will be discontinued from the study and replaced. Adverse events (AEs) and serious AEs (SAEs), including transfusion reactions (TRs) meeting Centers for Disease Control and Prevention (CDC) National Healthcare Safety Network (NHSN) definitions [23], and protocol-specified AEs, will be assessed from the start of surgery or the start of the first study RBC transfusion (whichever is first) daily and documented in electronic case report forms (eCRFs) and stored in an electronic data capture (EDC) system through post-operative day 28. AEs, SAEs, and TRs will not be analyzed for subjects that receive no study transfusions and are withdrawn from the study. The investigator will categorize AEs according to their relatedness, severity, seriousness, and expectedness. The Sponsor will categorize and analyze AEs using the Medical Dictionary for Regulatory Activities (MedDRA v.21, available at www.MedDra.org) dictionary published by the International Council for Harmonization.

Daily sCr assessments will be recorded up to and including 48 ± 4 h post-surgery. Other parameters will be derived (when available) from subjects’ medical records and entered in the EDC system.

Transfusions will be administered according to local institutional policy and safety standards as ordered by the medical team. When study RBCs are unavailable or a subject’s need for RBC transfusions exceeds the quantity of available study RBCs (e.g., during a massive transfusion protocol), non-study conventional RBCs may be transfused. Protocol-defined hemodynamic and hospital clinical laboratory test results will be recorded daily throughout the acute transfusion support period. If sCr is measured multiple times in a day, the highest daily value will be recorded. If a subject is discharged prior to the end of the acute transfusion support period but returns to the study site for any reason (e.g., re-hospitalization or routine post-operative care) within the 7-day post-surgery period, all routinely collected hospital clinical laboratory values will be recorded in the EDC system. For discontinued, non-transfused subjects, a blood sample for sCr will be drawn at 48 ± 4 h, but other post-baseline laboratory parameters and AE data will not be analyzed. Vital status will be recorded at the time of discontinuation. Urine output (mL/kg/hour) will be recorded in the EDC system daily while a urinary catheter is in place. Other assessments will capture details related to the specific surgical procedure, e.g., type of procedure, start and end surgical procedure times, duration of cardiac bypass, RBC components transfused, all other blood components transfused, estimated blood loss from the surgical procedure, concomitant medications, intraoperative RBC salvage and reinfusion, hemodilution, and nadir temperature. Daily estimated blood loss from chest tube(s) and from other sources will also be recorded. Subsequent post-operative assessments will only apply to randomized subjects who receive a study RBC transfusion.

#### Post-operative period (through day 28 after last study transfusion)

Following day 7 of the acute transfusion support period, subjects will receive conventional non-study RBCs if additional RBC transfusions are needed, as indicated by their treating physician. Weekly telephone surveillance calls to the subject will be performed to collect AE, SAE, and TR data. Laboratory results recorded in the EDC during this period will only be those assessed as clinically significant by the site investigator.

#### Day 28 ± 3 after last study transfusion or early termination

Subjects will be scheduled for a follow-up visit 28 ± 3 days after the last study transfusion. Blood samples for the determination of HLA antibodies will also be collected. Transfusion reactions, AEs, and SAEs will be documented for the full 28-day period after the last study transfusion. Subjects will have their vital status and need for renal replacement therapy (RRT) documented at day 30 after surgery.

#### End of study (75 ± 15 days after last study transfusion)

Subjects will be scheduled for a second follow-up visit on day 75 ± 15 days after the last study transfusion for vital status, need for RRT, and for assessment of amustaline/GSH RBC-specific antibodies at the end of study. Subjects will be on-study for a minimum of 75 days and a maximum of 127 days per protocol.

### Discontinuing study and stopping rules

Study subjects are free to withdraw consent or discontinue participation in the study at any time. Investigators may also terminate subjects’ participation in the study at any time without prejudice to further treatment. Study RBC transfusions will be discontinued if the subject becomes pregnant; is treated with a concurrent medication demonstrated to have caused hemolysis; develops an antibody with specificity for amustaline/GSH RBCs; or has an IAT finding that cannot rule out amustaline/GSH specific RBC antibodies.

The study may be temporarily paused, placed on clinical hold, or stopped based on poor/slow accrual; based on safety recommendations from the Data Safety Monitoring Board (DSMB); or if one subject demonstrates a confirmed amustaline/GSH RBC antibody associated with a clinically significant hemolytic TR that cannot be explained by other conditions. No subjects will be enrolled in the study during a clinical hold. Subjects already enrolled will be withdrawn from receiving study RBCs, pending consultation with the DSMB and FDA.

### Sample size

For the primary efficacy endpoint, a baseline AKI event rate of 30% will be assumed in both the Test and Control groups based on two prior Cerus-sponsored studies in transfused cardiac surgery patient populations similar to that proposed in ReCePI [8, 24]. A reanalysis of the primary data from these studies showed the proportions of Control patients that had ΔsCr values within 48 h of surgery completion as follows: ≥ 0.1 mg/dL = 55%; ≥ 0.2 mg/dL = 45%; and ≥ 0.3 mg/dL = 30%. Based on these data, Cerus proposed a non-inferiority design in ReCePI to rule out an increase of ΔsCr ≥ 0.3 mg/dL. The non-inferiority margin with the assumed 30% incidence in the Control group (50% of the Control rate) is 15%. A sample size of at least 292 patients (146 per arm) will provide approximately 80% power to declare non-inferiority at the two-sided 0.05 alpha level.

### Blinding

Operating room, surgical, and intensive care unit (ICU) staff, and all others caring for study subjects, as well as the Sponsor (and delegates) will be blinded to treatment assignment. Study RBC components will be labeled with an identical label for Test and Control components. Designated transfusion service staff and unblinded delegates who monitor the production of the RBC components will be able to access the treatment arm assignment to ensure the correct (Test or Control) study RBCs are issued. An unblinded study monitor will review and verify source data collected at the transfusion services. An investigator may request unblinding after consultation with the Sponsor on the basis of the need to treat subjects with AEs related to the study product. The DSMB will be notified in the event of any unblinding requests. Unblinding would be restricted to only those personnel that require the information to provide medical care to the subject.

### Data management

Subjects’ medical records, transfusion service electronic records, and blood center electronic records are the source data. All study data will be recorded on customized eCRFs stored in a proprietary EDC system (iMedidata RAVE, Medidata Solutions, New York, NY) developed by a contracted clinical research organization (CRO) (PPD, Inc. Wilmington, NC). Source data will be verified against the EDC system by the CRO. The Sponsor will perform data quality checks on blinded study data on a monthly basis. All study documents will be entered into an electronic trial master file (TMF) maintained by the Sponsor.

#### Confidentiality

Individual subject’s medical information obtained as a result of this study is considered confidential. Disclosure to third parties is prohibited. Subject confidentiality will be assured by identification code numbers which blinded study personnel will not be able to link to treatment assignment arm or any individual subject. Medical information may be given to subjects’ personal physicians, or to other appropriate medical personnel responsible for their welfare. Anonymized data generated as a result of this study will also be available for inspection upon request by health authorities, the Sponsor, the DSMB, or by IRBs.

### Outcomes

#### Primary and secondary endpoints

The primary efficacy endpoint is the proportion of patients who have received at least one study RBC transfusion and have a diagnosis of acute kidney injury defined as:

Any increased sCr level occurring after transfusion of a study RBC of ≥ 0.3 mg/dL (or 26.5 µmol/L) from the pre-surgery baseline within 48 ± 4 h of the end of surgery.

The primary safety endpoints are:

- The proportion of patients with any treatment-emergent adverse events (TEAEs) possibly, probably, or definitely related to study RBC transfusion through 28 days after the last study transfusion.
- The proportion of patients with treatment-emergent antibodies with confirmed specificity to amustaline/GSH RBCs by the end of study.

TEAEs will comprise all untoward medical events occurring after the start of the first study RBC transfusion and during the safety assessment period (i.e., AEs, SAEs, and TRs occurring within 28 days after the last study RBC transfusion).

Secondary endpoints include:

- The proportion of patients with a diagnosis of stage I, II, or III Acute Kidney Injury (KDIGO 2012) within 7 days of the end of surgery.
- Mortality or the need for RRT by 30 days post-surgery.
- Treatment-emergent immunization to RBC alloantigens.
- Treatment-emergent immunization to HLA alloantigens.

#### Statistical methods

The primary endpoints will be assessed in a modified intention-to-treat (mITT) population defined as all randomized subjects who receive at least one study RBC transfusion. Efficacy analyses will be summarized by treatment group assigned at randomization. Safety analyses will be summarized by the actual RBC treatment received. A per-protocol set (PPS) will be used for exploratory analysis and will be summarized by treatment group as randomized. Important protocol deviations that might affect the primary efficacy endpoint will be identified before unblinding and will be excluded from the PPS. Because the protocol allows for the transfusion of non-study RBCs when study RBCs are unavailable, patients who received study and non-study RBCs will be considered as having been transfused per protocol. Analyses will also be performed on a Study RBC Only Set (SROS) excluding subjects who received non-study RBC within 48 h of the completion of surgery.

The primary efficacy endpoint hypothesis is:

$${H}_{0}: {P}_{Test}-{P}_{Control}\ge 50\%\times {\widehat{P}}_{Control}$$

versus

$${H}_{A}:{ P}_{Test}-{P}_{Control}<50\%\times {\widehat{P}}_{Control}$$

where

${P}_{Test}$ and ${P}_{Control}$ are the event rates for Test and Control groups, respectively and ${\widehat{P}}_{Control}$ is the observed Control rate.

The primary analysis will be based on the Miettinen and Nurminen (M&N) method stratified by baseline sCr (sCr ≥ 1.2 mg/dL vs. < 1.2 mg/dL) and cardiac surgery group performed (more at risk for renal complications vs. less at risk) based on the observed cases. Non-inferiority will be claimed if the upper bound of the 2-sided Cochran-Mantel-Haenszel (CMH) Score 95% confidence interval (CI) for the treatment difference (Test – Control) is less than 50% of the observed Control rate. Superiority for the Test product may be claimed if the upper bound of the 95% CI is less than 0.

Sensitivity analyses will be performed to assess the robustness of the results of the primary analysis. To correct for the impact of switching from study RBCs to non-study RBCs in patients who receive both types of RBCs, an inverse probability censoring weighting method will be used. To derive weights adjusting for the confounding effects of non-study RBCs, both baseline and post-baseline covariates will be considered in this approach. In the multivariate logistic regression models used for weight calculations, selected covariates must have a *p*-value of ≤ 0.1.

Exploratory analysis may be conducted as suggested by the data. No formal statistical inferences will be drawn from exploratory analyses. The CMH test will also be used to assess treatment differences for the secondary efficacy endpoints. No formal statistical inferences will be drawn from the secondary efficacy analyses.

To assess the homogeneity of treatment effects, subgroup analyses with descriptive summaries will be performed by age groups (< 65 vs. ≥ 65 years), sex (males or females), race (White or Other [Black or African American, Asian, American Indian or Alaska Native, Native Hawaiian or Other Pacific Islander, Other]), baseline sCr (sCr ≥ 1.2 mg/dL vs. < 1.2 mg/dL), baseline TRUST score (< 3 vs. ≥ 3), and cardiac surgery group (more at risk for renal complications vs. less at risk) which will be reported for sCr and primary efficacy endpoint. Other subgroup analyses may be performed as suggested by the data.

A descriptive summary of each safety-related measure will be performed. The primary safety endpoints (related TEAEs and treatment-emergent antibodies with specificity to amustaline/GSH-treated RBCs) will be compared and tabulated by treatment using frequency (*n*) and percent (%). *P*-values from a CMH test stratified by baseline sCr (sCr ≥ 1.2 mg/dL vs. < 1.2 mg/dL) and cardiac surgery group performed (more at risk for renal complications vs. less at risk) will be presented in an exploratory sense only. Other safety endpoints, including RBC alloantibodies and HLA alloantibodies will be carried out in the same manner.

For the primary analysis of the primary efficacy endpoint, no imputation is needed and the analysis will be based on observed cases. If a subject has no post-baseline sCr available within 48 ± 4 h post-surgery, the subject will be treated as missing the primary endpoint. For the sensitivity analyses of the primary efficacy endpoint, missing data will be imputed using multiple imputation.

#### Interim analysis

A blinded analysis of the incidence of the primary endpoint was performed for sample size re-estimation by the Sponsor in October 2021 after ~ 200 subjects had been enrolled and transfused. Consultation with the FDA resulted in a reduction in the study’s sample size from 600 to ≥ 292 patients based on the measured pooled incidence of the primary endpoint and a re-estimation of the study power. No other interim analyses are planned.

### Conduct of the study

The study is coordinated, monitored, and steered by the Sponsor’s clinical operations and data management staff, under the direction of the Sponsor’s Chief Medical Officer. FDA-approved protocol amendments will be provided to the IRB governing each study site for local approval before implementation. The Sponsor will train study staff on protocol modifications before implementation. A clinical study report will be submitted to the US FDA and results will be available on www.clinicaltrials.gov within 12 months of completion of the study.

Study outcomes will be published in a peer-reviewed journal and disseminated through academic and commercial presentations. Investigators will share authorship. The participant-level data and statistical code will not be made generally available. Interested parties may approach the Sponsor for access on a case-by-case basis.

### Data and safety monitoring board

Study data and safety will be monitored by a DSMB that is independent of the Sponsor, funder (BARDA), and Investigators. The DSMB is composed of transfusion medicine, internal medicine, statistical, and other experts as described in the DSMB Charter (available on request from the Sponsor). The DSMB meets on an agreed timeline based on recruitment to review safety and efficacy data in a group-blinded fashion and may request unblinding of the data as needed to ensure subject safety.

## Discussion

The ReCePI study aims to demonstrate the safety and efficacy of amustaline/GSH pathogen-reduced RBCs in subjects treated for acute anemia related to bleeding where transfusions are indicated to prevent tissue hypoxia. The complex cardiac surgery patient population was selected as having a predictable need for RBC transfusions to replace surgical blood loss with increased susceptibility to the effects of tissue hypoxia due to the nature of the surgery, the use of cardiopulmonary bypass and generally older age [25]. The selection of the primary endpoint followed multiple discussions with the FDA, with a focus on demonstrating the physiologic function of transfused RBCs in vivo. There are no validated outcome measures of RBC efficacy that assess tissue oxygenation function. Clinically, RBC transfusion efficacy is monitored using hemoglobin increments, a surrogate measure, and transfusions are guided by “threshold” hemoglobin values or the extent of surgical blood loss. Recent studies demonstrate that restrictive threshold values reduce the use of RBC transfusions without negative effects on outcomes, implying that maintenance of higher hemoglobin levels serves little benefit [26, 27]. These studies do not prove the RBC transfusions are intrinsically beneficial. In bleeding patients, hemoglobin levels may not reflect tissue oxygenation as fluid shifts occur in parallel to transfusion. Prior clinical studies questioned whether older RBCs are equally safe and effective as fresh RBCs, utilizing endpoints such as a multiple organ dysfunction score (MODS) [25], mortality [28] or other combinations of morbidity and mortality [29, 30]. These outcomes were not favored by the FDA as endpoints for licensing studies to demonstrate the efficacy of pathogen-reduced RBCs. We selected an adaptation of the KDIGO definition [31] of AKI as a novel primary efficacy endpoint to assess RBC function on the basis that AKI is relatively common after complex cardiac surgery; is thought to result from insults that include tissue hypoxia; and is highly associated with poor long-term patient outcomes, including death and the need for RRT 30 days after surgery [32, 33]. Furthermore, interventions to improve renal oxygenation such as goal-directed oxygen delivery on cardiopulmonary bypass are recognized as key to prevent AKI in this setting. Interestingly, raising the RBC transfusion threshold above the currently widely accepted value of 7.5–8.0 g/dL does not prevent AKI [34]. The AKI outcome was defined using sCr within 48 h post-surgery and excluded the use of urinary volume and change in sCr over 7 days to focus on the combined effects of cardiac surgery and study RBC transfusion to the exclusion of other post-surgical conditions associated with AKI. ReCePI is powered on an expected 30% incidence of AKI in high-risk complex cardiac surgery subjects based on the incidence of renal impairment in two prior studies performed by the Sponsor [8, 24]. Given the uncertainty in the incidence in a highly selected group of CVS patients, the non-inferiority margin was defined based on a proportion (50%) of the Control group rate rather than on a fixed incidence target. With a Control group rate of 30% AKI, the non-inferiority margin would be a rate increase of 15% in the Test group.

The primary efficacy endpoint of ReCePI will be assessed in a mITT analysis set, including subjects who are randomized and receive study RBCs within 48 h of the end of surgery. The protocol recognizes that due to the unpredictable requirement for massive transfusions that may exceed participating transfusion services’ capacity to provide study RBCs in cases with massive bleeding, non-study RBCs may need to be provided. Every effort is being made to provide adequate amounts and equal proportions of study RBCs; however, this approach has led to high wastage rate of study RBCs. The statistical analysis plan includes an exploratory analysis of subjects that receive study RBCs only (SRO set); however, the protocol is not powered for this analysis.

An important secondary endpoint is the incidence and clinical significance of antibodies to neoantigens formed as a result of amustaline and/or GSH binding to RBC surface membrane proteins or lipids during the pathogen reduction process. Treatment-emergent antibodies specific to amustaline/GSH RBCs were observed with a predicate pathogen reduction device [8] and could be neutralized with acridine in solution, thereby defining specificity for the acridine moiety of the amustaline molecule (also known as S-303). The antibodies were not associated with any clinical AEs. Geisen et al. [19] reported the prevalence of natural antibodies with specificity for amustaline/GSH RBCs. Five plasma samples from 998 subjects requiring chronic transfusion support for anemia and 12 plasma samples from 10,721 general hospital subjects never exposed to amustaline/GSH RBC demonstrated low titer reactivity with amustaline/GSH RBCs. The cumulative prevalence of natural antibodies to amustaline/GSH RBCs was 0.15% in this study. All antibodies were low titer (< 1:32) and were of the IgM and/or IgG subclass. None were found to be IgG_1_ or IgG_3_ subclass, the antibody subclasses most associated with physiologic hemolytic activity. The majority demonstrated specificity for acridine [19]. The ReCePI study is designed to exclude subjects with natural antibodies to amustaline/GSH RBCs at baseline and to detect and thoroughly investigate the clinical significance of treatment-emergent antibodies. The occurrence of a single subject with evidence of overt hemolysis associated with an amustaline/GSH antibody would require a clinical hold with evaluation and DSMB and FDA approval to continue enrolment. No hemolytic antibodies have been reported at the time of this publication and none have been observed in any previous or on-going clinical study with amustaline/GSH RBCs. A small number of non-clinically significant, low titer antibodies have been detected in the ReCePI study to date that resemble the natural antibodies described by Geisen et al. [19]. The study remains blinded and it is not known whether those subjects received Test or Control study RBCs.

Other secondary endpoints include the incidence of HLA antibodies, and mortality and renal replacement therapy (RRT) 28–30 days after the last study transfusion. Thirty-day mortality and RRT have been associated with the incidence of AKI within 48 h of surgery [32, 33, 35] and will be assessed in this study population. While treatment-emergent HLA antibodies are likely to be reduced by the use of leukocyte-reduced RBCs in both Test and Control arms, we hypothesize that inactivation of residual leukocytes by the amustaline/GSH pathogen reduction process might affect the incidence of alloimmunization.

## Trial status

ReCePI opened for enrollment at 18 US sites and transfused the first subject in January 2019. The current protocol (CLI 00125 version 8.0) incorporates revisions to improve comprehension and a reduction in the total number of transfused subjects from 600 to ≥ 292 subjects following a blinded interim analysis that confirmed the overall incidence of the primary endpoint and the power of the study. Enrollment was severely impacted by the COVID-19 pandemic between 2020 and 2022. In view of robust patient blood management practices and despite stringent selection criteria, only ~ 56% of enrolled subjects were transfused (site range 18–82%). The study is expected to be completed by the end of 2023 with ~ 620 subjects enrolled and ~ 320 subjects transfused.

### Supplementary Information

**Additional file 1. **SPIRIT Checklist for Trials.

## Data Availability

Consent forms and trial data will be available upon request from the Sponsor. The Protocol and statistical analysis plan will be published along with the clinical outcomes of the study on www.clinicaltrials.gov.

## Abbreviations

AE: Adverse event
AKI: Acute kidney injury
BARDA: Biological Advanced Research and Development Authority
CDC: Centers for Disease Control and Prevention
CPB: Cardiopulmonary bypass
CRO: Clinical research organization
CVS: Cardiovascular surgery
DAT: Direct antiglobulin test
DSMB: Data and Safety Monitoring Board
EDC: Electronic data capture
eCRF: Electronic case report forms
EDQM: European Directorate for the Quality of Medicines & Healthcare
FDA: Food and Drug Administration
GCP: Good Clinical Practice
GSH: Glutathione
HLA: Human leukocyte antigen
IAT: Indirect antiglobulin test
IRB: Institutional review board
IWRS: Interactive Web Response System
mITT: Modified intention-to-treat
NHSN: National Healthcare Safety Network
PPS: Per-protocol set
PRT: Pathogen reduction technology
RBC: Red blood cell
S-303: Amustaline
SAE: Serious adverse event
SCD: Sickle cell disease
sCr: Serum creatinine
SROS: Study red blood cells only set
RRT: Renal replacement therapy
TTI: Transfusion-transmitted infection
TA-GVHD: Transfusion-associated graft-versus-host disease
TEAE: Transfusion emergent adverse event
TR: Transfusion reaction
TRALI: Transfusion-related acute lung injury

## References

[1] Godbey EA, Thibodeaux SR (2019). Ensuring safety of the blood supply in the United States: Donor screening, testing, emerging pathogens, and pathogen inactivation. Semin Hematol.

[2] Stramer SL (2014). Current perspectives in transfusion-transmitted infectious diseases: emerging and re-emerging infections. ISBT Sci Ser.

[3] Haass KA, Sapiano MRP, Savinkina A, Kuehnert MJ, Basavaraju SV (2019). Transfusion-Transmitted Infections Reported to the National Healthcare Safety Network Hemovigilance Module. Transfus Med Rev.

[4] Gallian P, Pouchol E, Djoudi R, Lhomme S, Mouna L, Gross S (2019). Transfusion-Transmitted Hepatitis E Virus Infection in France. Transfus Med Rev.

[5] Kopolovic I, Ostro J, Tsubota H, Lin Y, Cserti-Gazdewich CM, Messner HA (2015). A systematic review of transfusion-associated graft-versus-host disease. Blood.

[6] Corash L, Benjamin RJ (2016). The role of hemovigilance and postmarketing studies when introducing innovation into transfusion medicine practice: the amotosalen-ultraviolet A pathogen reduction treatment model. Transfusion.

[7] Mufti NA, Erickson AC, North AK, Hanson D, Sawyer L, Corash LM (2010). Treatment of whole blood (WB) and red blood cells (RBC) with S-303 inactivates pathogens and retains in vitro quality of stored RBC. Biologicals.

[8] Benjamin RJ, McCullough J, Mintz PD, Snyder E, Spotnitz WD, Rizzo RJ (2005). Therapeutic efficacy and safety of red blood cells treated with a chemical process (S-303) for pathogen inactivation: a Phase III clinical trial in cardiac surgery patients. Transfusion.

[9] Henschler R, Seifried E, Mufti N (2011). Development of the S-303 Pathogen Inactivation Technology for Red Blood Cell Concentrates. Transfus Med Hemother.

[10] Castro G, Stassinopoulos A (2016). Effective Inactivation of T-Cells with Amustaline/GSH in Human RBC as assessed by Fluorescent Limiting Dilution Assays (LDA). Transfusion Clinique et Biologique..

[11] European Directorate for the Quality of Medicines and Healthcare (EDQM). Guide to the preparation, use and quality assurance of blood components Strasbourg. France: EDQM; 2023. Available from: https://freepub.edqm.eu/publications/10/detail. Accessed 4 Dec 2023.

[12] Cancelas JA, Gottschall JL, Rugg N, Graminske S, Schott MA, North A (2017). Red blood cell concentrates treated with the amustaline (S-303) pathogen reduction system and stored for 35 days retain post-transfusion viability: results of a two-centre study. Vox Sang.

[13] Benjamin RJ, Braschler T, Weingand T, Corash LM (2017). Hemovigilance monitoring of platelet septic reactions with effective bacterial protection systems. Transfusion.

[14] Knutson F, Osselaer J, Pierelli L, Lozano M, Cid J, Tardivel R (2015). A prospective, active haemovigilance study with combined cohort analysis of 19,175 transfusions of platelet components prepared with amotosalen-UVA photochemical treatment. Vox Sang.

[15] Cancelas J, Gottschall J, Rugg N, Graminske S, Schott M, North A (2015). Red Blood Cells (RBC) Treated with the INTERCEPT Pathogen and Leukocyte Inactivation System and Stored for 35 Days Retain Viability. Transfusion.

[16] Brixner V, Leibacher J, Pfeiffer H-U, Muller M, Geisen C, Henschler R (2015). Red Blood Cells Treated With the S-303 System for Pathogen Inactivation Demonstrate In Vitro Characteristics Suitable for Transfusion - Phase III Clinical Trial in Cardiac Surgery Patients. Vox sanguinis..

[17] Aydinok Y, Piga A, Origa R, Mufti N, Erickson A, North A (2019). Amustaline-glutathione pathogen-reduced red blood cell concentrates for transfusion-dependent thalassaemia. Br J Haematol.

[18] Rico S, North A, Lin JS, Corash L, NM (2015). Cumulative Clinical Safety Experience In Acute Anemia Transfusion Support With the INTERCEPT System for Red Blood Cells (RBC). Vox Sang..

[19] Geisen C, North A, Becker L, Brixner V, von Goetz M, Corash L (2020). Prevalence of natural and acquired antibodies to amustaline/glutathione pathogen reduced red blood cells. Transfusion.

[20] Garratty G (2012). What is a clinically significant antibody?. ISBT Sci Ser.

[21] Chan AW, Tetzlaff JM, Gøtzsche PC, Altman DG, Mann H, Berlin JA (2013). SPIRIT 2013 explanation and elaboration: guidance for protocols of clinical trials. BMJ.

[22] Alghamdi AA, Davis A, Brister S, Corey P, Logan A (2006). Development and validation of Transfusion Risk Understanding Scoring Tool (TRUST) to stratify cardiac surgery patients according to their blood transfusion needs. Transfusion.

[23] Centers for Disease Control and Prevention (2023). The National Healthcare Safety Network Biovigilance Component Hemovigilance Module Surveillance Protocol.

[24] Brixner V, Kiessling AH, Madlener K, Muller MM, Leibacher J, Dombos S (2018). Red blood cells treated with the amustaline (S-303) pathogen reduction system: a transfusion study in cardiac surgery. Transfusion.

[25] Steiner ME, Ness PM, Assmann SF, Triulzi DJ, Sloan SR, Delaney M (2015). Effects of red-cell storage duration on patients undergoing cardiac surgery. N Engl J Med.

[26] Hebert PC, Wells G, Blajchman MA, Marshall J, Martin C, Pagliarello G (1999). A multicenter, randomized, controlled clinical trial of transfusion requirements in critical care. Transfusion Requirements in Critical Care Investigators, Canadian Critical Care Trials Group. N Engl J Med..

[27] Carson JL, Carless PA, Hebert PC (2012). Transfusion thresholds and other strategies for guiding allogeneic red blood cell transfusion. Cochrane Database Syst Rev..

[28] Lacroix J, Hebert PC, Fergusson DA, Tinmouth A, Cook DJ, Marshall JC (2015). Age of transfused blood in critically ill adults. N Engl J Med.

[29] Fergusson DA, Hebert P, Hogan DL, LeBel L, Rouvinez-Bouali N, Smyth JA (2012). Effect of fresh red blood cell transfusions on clinical outcomes in premature, very low-birth-weight infants: the ARIPI randomized trial. JAMA.

[30] Hebert PC, Chin-Yee I, Fergusson D, Blajchman M, Martineau R, Clinch J (2005). A pilot trial evaluating the clinical effects of prolonged storage of red cells. Anesth Analg.

[31] KDIGO. Kidney Disease: Improving Global Outcomes (KDIGO) Acute Kidney Injury Work Group (2012). KDIGO Clinical Practice Guideline for Acute Kidney Injury. Kidney Inter Suppl.

[32] Lassnigg A, Schmid ER, Hiesmayr M, Falk C, Druml W, Bauer P (2008). Impact of minimal increases in serum creatinine on outcome in patients after cardiothoracic surgery: do we have to revise current definitions of acute renal failure?. Crit Care Med.

[33] Lassnigg A, Schmidlin D, Mouhieddine M, Bachmann LM, Druml W, Bauer P (2004). Minimal changes of serum creatinine predict prognosis in patients after cardiothoracic surgery: a prospective cohort study. J Am Soc Nephrol.

[34] Peng K, McIlroy DR, Bollen BA, Billings FTt, Zarbock A, Popescu WM (2022). Society of Cardiovascular Anesthesiologists Clinical Practice Update for Management of Acute Kidney Injury Associated With Cardiac Surgery. Anesth Analg..

[35] Bernardi MH, Schmidlin D, Schiferer A, Ristl R, Neugebauer T, Hiesmayr M (2015). Impact of preoperative serum creatinine on short- and long-term mortality after cardiac surgery: a cohort study. Br J Anaesth.

