# Supplementary material for: Evaluation of the efficacy and safety of amustaline/glutathione pathogen-reduced RBCs in complex cardiac surgery: the Red Cell Pathogen Inactivation (ReCePI) study—protocol for a phase 3, randomized, controlled trial
Source: Trials. 2023 Dec 11;24:799. doi: 10.1186/s13063-023-07831-x (PMC10712151; doi:10.1186/s13063-023-07831-x)
Supplement: Supplementary file 1 — Additional file 1. SPIRIT Checklist for Trials. [file 13063_2023_7831_MOESM1_ESM.pdf]

SPIRIT Checklist for *Trials*

Complete this checklist by entering the page and line numbers where each of the items listed below can be found in your manuscript.

Your manuscript may not currently address all the items on the checklist. Please modify your text to include the missing information. If you are certain that an item does not apply, please state "n/a" and provide a short explanation. **Leaving an item blank or stating "n/a" without an explanation will lead to your manuscript being returned before review.**

Upload your completed checklist as an additional file when you submit to *Trials*. You must reference this additional file in the main text of your protocol submission. The completed SPIRIT figure must be included within the main body of the protocol text and can be downloaded here: http://www.spirit-statement.org/schedule-of-enrolment-interventions-and-assessments/

In your methods section, please state that you used the SPIRIT reporting guidelines, and cite them as:

Chan A-W, Tetzlaff JM, Gøtzsche PC, Altman DG, Mann H, Berlin J, Dickersin K, Hróbjartsson A, Schulz KF, Parulekar WR, Krleža-Jerić K, Laupacis A, Moher D. SPIRIT 2013 Explanation and Elaboration: Guidance for protocols of clinical trials. BMJ. 2013;346:e7586

		Reporting Item	Page and Line Number	Reason if not applicable
**Administrative information**				
Title	#1	Descriptive title identifying the study design, population, interventions, and, if applicable, trial acronym	Page 1, lines 1-3 and pg. 4, lines 95-97]	
Trial registration	#2a	Trial identifier and registry name. If not yet registered, name of intended registry	Page 4, lines 98-100]	
Trial registration: data set	#2b	All items from the World Health Organization Trial Registration Data Set	n/a]	Registration includes most elements of the WHO registration data set, however the completion date and summary of results are not yet available. Final full data set will

				be posted on www.clinicaltrials.gov within 12 months of finalization.]
Protocol version	#3	Date and version identifier	Page 4, line 101]	
Funding	#4	Sources and types of financial, material, and other support	Page 5, lines 102-104]	
Roles and responsibilities: contributorship	#5a	Names, affiliations, and roles of protocol contributors	Names & affiliations: Pages 1-3, lines 4-56. Roles & responsibilities: Page 21, lines 591-597]	
Roles and responsibilities: sponsor contact information	#5b	Name and contact information for the trial sponsor	Page 5, lines 109-114]	
Roles and responsibilities: sponsor and funder	#5c	Role of study sponsor and funders, if any, in study design; collection, management, analysis, and interpretation of data; writing of the report; and the decision to submit the report for publication, including whether they will have ultimate authority over any of these activities	Page 5, lines 115-117]	
Roles and responsibilities: committees	#5d	Composition, roles, and responsibilities of the coordinating centre, steering committee, endpoint adjudication committee, data management team, and other individuals or groups overseeing the trial, if applicable (see Item 21a for data monitoring committee)	n/a]	The DSMB (described in item 21a) is the only committee associated with this study protocol; the data management team is comprised of Sponsor staff and CRO contractors; these details are not included in the protocol. No coordinating center, steering committee or endpoint adjudication committee utilized.]

**Introduction**			Pages 6-8, lines 153-203]	
Background and rationale	#6a	Description of research question and justification for undertaking the trial, including summary of relevant studies (published and unpublished) examining benefits and harms for each intervention	Pages 6-8, lines 153-203]	
Background and rationale: choice of comparators	#6b	Explanation for choice of comparators	Pages 9-10, lines 241-251]	
Objectives	#7	Specific objectives or hypotheses	Page 8, lines 205-215]	
Trial design	#8	Description of trial design including type of trial (eg, parallel group, crossover, factorial, single group), allocation ratio, and framework (eg, superiority, equivalence, non-inferiority, exploratory)	Page 8, lines 205-210]	
**Methods: Participants, interventions, and outcomes**				
Study setting	#9	Description of study settings (eg, community clinic, academic hospital) and list of countries where data will be collected. Reference to where list of study sites can be obtained	Page 9, lines 224-226]	
Eligibility criteria	#10	Inclusion and exclusion criteria for participants. If applicable, eligibility criteria for study centres and individuals who will perform the interventions (eg, surgeons, psychotherapists)	Page 9, lines 227-239 and Table 1, page 27, line 715]	

Interventions: description	#11a	Interventions for each group with sufficient detail to allow replication, including how and when they will be administered	Page 9, lines 241-246 and Page 11, 277-279]	
Interventions: modifications	#11b	Criteria for discontinuing or modifying allocated interventions for a given trial participant (eg, drug dose change in response to harms, participant request, or improving / worsening disease)	Pages 12-13, lines 334-346]	
Interventions: adherance	#11c	Strategies to improve adherence to intervention protocols, and any procedures for monitoring adherence (eg, drug tablet return; laboratory tests)	Page 13, line 358-359 and Page 14, lines 365-371]	
Interventions: concomitant care	#11d	Relevant concomitant care and interventions that are permitted or prohibited during the trial	Page 11, lines 275-316 and Table 1, page 27, line 715]	
Outcomes	#12	Primary, secondary, and other outcomes, including the specific measurement variable (eg, systolic blood pressure), analysis metric (eg, change from baseline, final value, time to event), method of aggregation (eg, median, proportion), and time point for each outcome. Explanation of the clinical relevance of chosen efficacy and harm outcomes is strongly recommended	Pages 14-15, lines 381-400 and Pages 18-19, lines 501-517.]	
Participant timeline	#13	Time schedule of enrolment, interventions (including any run-ins and washouts), assessments, and visits for participants. A schematic diagram is highly recommended (see Figure)	Pages 10-16, lines 253-333, and Figure 1, page 30, line 733]	

Sample size	#14	Estimated number of participants needed to achieve study objectives and how it was determined, including clinical and statistical assumptions supporting any sample size calculations	Page 13, lines 347-351]	
Recruitment	#15	Strategies for achieving adequate participant enrolment to reach target sample size	Page 10, lines 257-260]	
**Methods: Assignment of interventions (for controlled trials)**				
Allocation: sequence generation	#16a	Method of generating the allocation sequence (eg, computer-generated random numbers), and list of any factors for stratification. To reduce predictability of a random sequence, details of any planned restriction (eg, blocking) should be provided in a separate document that is unavailable to those who enrol participants or assign interventions	Page 10, lines 269-274]	
Allocation concealment mechanism	#16b	Mechanism of implementing the allocation sequence (eg, central telephone; sequentially numbered, opaque, sealed envelopes), describing any steps to conceal the sequence until interventions are assigned	Page 10, lines 269-270]	
Allocation: implementation	#16c	Who will generate the allocation sequence, who will enrol participants, and who will assign participants to interventions	Page 10, lines 269-274]	
Blinding (masking)	#17a	Who will be blinded after assignment to interventions (eg, trial participants, care	Pages 13, lines 353-359]	

		providers, outcome assessors, data analysts), and how		
Blinding (masking): emergency unblinding	#17b	If blinded, circumstances under which unblinding is permissible, and procedure for revealing a participant's allocated intervention during the trial	Pages 13-14, lines 359-363]	
**Methods: Data collection, management, and analysis**				
Data collection plan	#18a	Plans for assessment and collection of outcome, baseline, and other trial data, including any related processes to promote data quality (eg, duplicate measurements, training of assessors) and a description of study instruments (eg, questionnaires, laboratory tests) along with their reliability and validity, if known. Reference to where data collection forms can be found, if not in the protocol	Page 14, lines 364-371]	
Data collection plan: retention	#18b	Plans to promote participant retention and complete follow-up, including list of any outcome data to be collected for participants who discontinue or deviate from intervention protocols	n/a (participant retention) and Pages 11-12, lines 303-308 and page 15, lines 405-412 (protocol deviations)]	There are no plans to promote participant retention (subjects may withdraw from the study if/when they choose, pg. 13, line 335). Telephone interviews (e.g., pg. 12, line 320) may be conducted to facilitate follow-up for subjects who may be unable to return to the study site.]
Data management	#19	Plans for data entry, coding, security, and storage, including any related processes to promote data quality (eg, double data entry; range checks for data values). Reference to	Page 17, lines 367-371]	

		where details of data management procedures can be found, if not in the protocol		
Statistics: outcomes	#20a	Statistical methods for analysing primary and secondary outcomes. Reference to where other details of the statistical analysis plan can be found, if not in the protocol	Pages 15-17, lines 401-433]	
Statistics: additional analyses	#20b	Methods for any additional analyses (eg, subgroup and adjusted analyses)	Page 16, lines 438-451	
Statistics: analysis population and missing data	#20c	Definition of analysis population relating to protocol non-adherence (eg, as randomised analysis), and any statistical methods to handle missing data (eg, multiple imputation)	Page 15, line 405-412, page 16, lines 438-444 (analysis populations), and Page 17, lines 452-456 (data imputation).	
**Methods: Monitoring**				
Data monitoring: formal committee	#21a	Composition of data monitoring committee (DMC); summary of its role and reporting structure; statement of whether it is independent from the sponsor and competing interests; and reference to where further details about its charter can be found, if not in the protocol. Alternatively, an explanation of why a DMC is not needed	Pages 17-18, lines 474-480]	
Data monitoring: interim analysis	#21b	Description of any interim analyses and stopping guidelines, including who will have access to these interim results and make the final decision to terminate the trial	Page 17, lines 457-462]	

Harms	#22	Plans for collecting, assessing, reporting, and managing solicited and spontaneously reported adverse events and other unintended effects of trial interventions or trial conduct	Pages 11-12, lines 282-293]	
Auditing	#23	Frequency and procedures for auditing trial conduct, if any, and whether the process will be independent from investigators and the sponsor	Page 13, lines 358-359 (unblinded blood bank monitoring) and Page 14, lines 368-370 (source data verification)	
**Ethics and dissemination**				
Research ethics approval	#24	Plans for seeking research ethics committee / institutional review board (REC / IRB) approval	Page 21, lines 569-572]	
Protocol amendments	#25	Plans for communicating important protocol modifications (eg, changes to eligibility criteria, outcomes, analyses) to relevant parties (eg, investigators, REC / IRBs, trial participants, trial registries, journals, regulators)	Page 17, lines 465-468]	
Consent or assent	#26a	Who will obtain informed consent or assent from potential trial participants or authorised surrogates, and how (see Item 32)	Page 9, lines 230-233]	
Consent or assent: ancillary studies	#26b	Additional consent provisions for collection and use of participant data and biological specimens in ancillary studies, if applicable	Page 9, lines 232-233]	
Confidentiality	#27	How personal information about potential and enrolled participants will be collected, shared,	Page 14, lines 372-379]	

		and maintained in order to protect confidentiality before, during, and after the trial		
Declaration of interests	#28	Financial and other competing interests for principal investigators for the overall trial and each study site	Page 21, lines 581-585]	
Data access	#29	Statement of who will have access to the final trial dataset, and disclosure of contractual agreements that limit such access for investigators	Page 21, lines 577-579 (data published on clinicaltrials.gov) and Page 17, lines 470-473 (access to data on request)]	
Ancillary and post trial care	#30	Provisions, if any, for ancillary and post-trial care, and for compensation to those who suffer harm from trial participation	n/a]	Patients will receive routine post-trial care as prescribed by their physicians; the Sponsor will not provide ancillary or post-trial care. Risks associated with participation in this trial are deemed to be minimal; no compensation for subjects who suffer harm is planned.]
Dissemination policy: trial results	#31a	Plans for investigators and sponsor to communicate trial results to participants, healthcare professionals, the public, and other relevant groups (eg, via publication, reporting in results databases, or other data sharing arrangements), including any publication restrictions	Page 17, lines 470-473]	
Dissemination policy: authorship	#31b	Authorship eligibility guidelines and any intended use of professional writers	Pages 17, line 471 (authorship);	

			professional writer(s) will not be used.]	
Dissemination policy: reproducible research	#31c	Plans, if any, for granting public access to the full protocol, participant-level dataset, and statistical code	Page 17, line 471-473 and page 21, line 576-579]	
**Appendices**				
Informed consent materials	#32	Model consent form and other related documentation given to participants and authorised surrogates	Page 21, line 577]	
Biological specimens	#33	Plans for collection, laboratory evaluation, and storage of biological specimens for genetic or molecular analysis in the current trial and for future use in ancillary studies, if applicable	n/a]	The current protocol does not include plans for genetic or molecular analyses on biological specimens. Additional consent will be sought from patients should any ancillary studies be planned with archived specimens (Page 9, lines232-234).

It is strongly recommended that this checklist be read in conjunction with the SPIRIT 2013 Explanation & Elaboration for important clarification on the items. Amendments to the protocol should be tracked and dated. The SPIRIT checklist is copyrighted by the SPIRIT Group under the Creative Commons "Attribution-NonCommercial-NoDerivs 3.0 Unported" license. This checklist can be completed online using https://www.goodreports.org/, a tool made by the EQUATOR Network in collaboration with Penelope.ai
